# Timing of planned reoperation after damage control surgery in patients with trauma: a systematic review and meta-analysis

**DOI:** 10.1186/s13017-025-00657-9

**Published:** 2025-10-29

**Authors:** Dongmin Seo, Hye Young Woo, Inhae Heo, Kyoungwon Jung, Hohyung Jung

**Affiliations:** 1 Division of Trauma Surgery, Department of Surgery, Ajou University School of Medicine, 164 Worldcup-ro, Yeongtong-gu, Suwon, Gyeonggi-do 16499 Republic of Korea; 2 Regional Trauma Centre of Southern Gyeong-gi Province, Ajou University School of Medicine, Suwon, Republic of Korea

**Keywords:** Damage control surgery, Planned reoperation, Reoperation timing, Trauma, Re-bleeding, Meta-analysis

## Abstract

**Background:**

Damage control surgery (DCS) is the standard approach for managing severely injured patients with trauma who present with extreme physiological derangements. The optimal timing for planned reoperation after the initial DCS remains contentious. Although traditional guidelines recommend reoperation within 24–48 h, emerging evidence suggests this interval may not be appropriate for all patients. This systematic review and meta-analysis evaluated the impact of early versus delayed planned reoperations on the clinical outcomes in patients with trauma following DCS.

**Methods:**

This review adhered to the Preferred Reporting Items for Systematic Reviews and Meta-Analyses (PRISMA) 2020 guidelines (PROSPERO registration: CRD420251049990). PubMed, Embase, and the Cochrane Library were searched from inception to 28 July 2025. Eligible studies compared early (≤ 48 h) with delayed (> 48 h) planned reoperation after DCS in adult patients with trauma. The primary outcome was re-bleeding; secondary outcomes were in-hospital mortality and infection rates. Study quality was assessed using the Newcastle–Ottawa Scale, and the certainty of evidence was graded using the GRADE approach. Meta-analysis was conducted using random-effects models.

**Results:**

Seven retrospective cohort studies involving 965 patients met the inclusion criteria. No prospective or randomised controlled trials were identified. Early planned reoperation was associated with significantly higher re-bleeding rates (OR 3.01; 95% CI 1.21–7.51; *P* = 0.02), indicating three-fold higher odds of re-bleeding with early intervention compared to delayed reoperation. No significant differences were observed in mortality (OR 0.79; 95% CI 0.51–1.23; *P* = 0.29; I^2^ = 0%) or infection rates (OR 1.05; 95% CI 0.54–2.05; *P* = 0.89; I^2^ = 65%).

**Conclusions:**

Delayed planned reoperation beyond 48 h after DCS significantly reduces the risk of re-bleeding, without increasing mortality or infection rates. These findings support an individualised approach to reoperation timing guided by patient physiology, rather than rigid adherence to conventional 24- to 48-h protocols.

**Supplementary Information:**

The online version contains supplementary material available at 10.1186/s13017-025-00657-9.

## Background

Damage control surgery (DCS) has transformed the management of critically ill, injured patients with trauma by prioritising rapid physiological stabilisation over definitive repair [1, 2]. This staged strategy involves prompt control of haemorrhage and contamination, temporary abdominal closure, often with gauze packing, and subsequent intensive care unit (ICU) resuscitation to address the lethal triad of hypothermia, acidosis, and coagulopathy [3, 4].

Originally developed in response to poor outcomes from prolonged surgery in physiologically unstable patients, DCS has markedly reduced mortality, particularly in those with exsanguinating injuries at non-compressible sites such as the thorax, abdomen, and pelvic cavities [5–8]. Following the initial DCS, a planned reoperation is typically performed to remove packing, reassess injuries, and achieve definitive anatomical repair or delayed fascial closure.

Although widely adopted, the optimal timing for planned reoperation remains controversial. Traditional recommendations advocate reoperation within 24–48 h after the initial DCS. These are based on the premise that earlier reoperation may precipitate re-bleeding because of unstable haemostasis, whereas excessive delays could increase infectious complications from retained contaminants or necrotic tissue [8, 9]. However, such guidance is largely derived from expert opinions or limited retrospective studies and may not represent best practices across different patient populations [10].

Recent evidence has questioned the universal applicability of the conventional 24- to 48-h window. While some studies suggest that reoperation timing may not significantly influence outcomes, others emphasise the need for patient-specific strategies guided by physiological recovery [11, 12]. Given the scarcity of high-quality data and the potential implications for clinical practice, we conducted a systematic review and meta-analysis to synthesise the current evidence on the timing of planned reoperation following DCS. This study aimed to inform clinical decision-making and guide future research towards more tailored reoperation protocols.

## Methods

### Study protocol and registration

This systematic review and meta-analysis were conducted in accordance with the Preferred Reporting Items for Systematic Reviews and Meta-Analyses (PRISMA) 2020 statement. The protocol was prospectively registered in the International Prospective Register of Systematic Reviews (PROSPERO) database (registration number: CRD420251049990).

### Eligibility criteria

The eligibility criteria were defined using the PICOS framework.

*Population*: Adult patients with trauma who underwent DCS for truncal injuries involving the thoracic, abdominal, or pelvic regions.

*Intervention*: Early planned reoperation, defined as ≤ 48 h after the initial DCS.

*Comparator*: Delayed planned reoperation, defined as > 48 h after the initial DCS.

*Outcomes*: Studies reporting at least one of the following: re-bleeding rate (primary outcome), in-hospital mortality, or infection rate (secondary outcome).

*Study design*: Randomised controlled trials, cohort studies, and case-control studies. Studies published in English were eligible. The 48-h threshold was selected based on: (1) the expected physiological correction of the lethal triad [6, 7]; (2) World Society of Emergency Surgery (WSES) Eastern Association for the Surgery of Trauma (EAST) guidelines [8, 9]; and (3) methodological consistency with prior studies [11–13].

### Search strategy

A comprehensive search was conducted in PubMed, Embase, and the Cochrane Library from their inception to 28 July 2025. The strategy combined Medical Subject Headings (MeSH), Emtree terms, and free-text keywords, covering three main concepts: (1) damage control surgery, (2) timing of planned reoperation, and (3) clinical outcomes in patients with trauma. The complete search strategies are provided in Supplementary Digital Content 1. No language restrictions were applied during the search; however, only English and Korean publications were screened, and all included studies were ultimately published in English. The final search update was performed on 28 July 2025.

### Study selection and data extraction

Following PRISMA 2020 guidelines, two reviewers (D.S., H.Y.W.) independently screened titles and abstracts against the predefined eligibility criteria. Full-text articles were then reviewed to confirm inclusion. Data extraction was conducted by three reviewers (D.S., H.Y.W., and I.H.) using a standardised data collection form. Any discrepancies were resolved by discussion and consensus.

### Definitions

The timing of planned reoperation was defined as the interval from the initial DCS to either formal reoperation or gauze pack removal. Studies not strictly applying the 48-h cut-off were included if patients could be categorised into early or delayed groups. Infection outcomes encompassed intra-abdominal infections (including abscesses and fluid collections), surgical site infections, and clinically diagnosed sepsis. Patients with non-abdominal and non-infectious complications were excluded.

### Quality assessment

The methodological quality of included studies was assessed using the Newcastle-Ottawa Scale (NOS) for cohort studies. The certainty of evidence for each outcome was graded using the Grading of Recommendations Assessment, Development and Evaluation (GRADE) approach (Tables 1 and 2).

**Table 1:** Data regarding the Newcastle–Ottawa quality assessment scale for assessing quality of the non-randomized studies

Study	Selection				Comparability	Outcome			Total score
	Representativeness of the exposed cohort	Selection of the non exposed cohort	Ascertainment of exposure	Demonstration that outcome of interest was not present at start of study	Comparability of cohorts on the basis of the design or analysis	Assessment of outcome	Was follow-up long enough for outcomes to occur	Adequacy of follow up of cohorts	
Jeong et al.	*	*	*	*	*	*	*	*	8/9
Ordonez et al.	*		*	*	*	*	*	*	7/9
Kang et al.	*	*	*	*	*	*	*	*	8/9
Nicol et al.	*	*	*	*	*	*	*	*	8/9
Caruso et al.	*		*	*		*	*	*	6/9
Pommerening et al.	*	*	*	*	*	*	*	*	8/9
Kim et al.	*	*	*	*	*	*			6/9

**Table 2:** The GRADE tool assessment

No of Study	Certainty assessment						Effect			Certainty	Importance
	Study design	Risk of bias	Inconsistency	Indirectness	Imprecision	Other considerations	No of events	No of individuals	Rate (95% CI)		
Rebleeding											
3	Non-randomised studies	Not serious	Not serious	Not serious	Not serious	Large effect	34	214	OR 3.01 (95% CI 1.21–7.51)	⨁⨁⨁◯ Moderate	Critical
In-hospital mortality											
4	Non-randomised studies	Not serious	Not serious	Not serious	serious	None	119	786	OR 0.79 (95% CI 0.51–1.23)	⨁⨁◯◯ Low	Critical
Infection											
6	Non-randomised studies	Not serious	Serious	Not serious	serious	None	379	858	OR 1.05 (95% CI 0.54–2.05)	⨁◯◯◯ Very low	Important

Participant numbers are outcome-specific and correspond to the studies reporting data for that outcome

### Statistical analysis

Pooled odds ratios (ORs) and 95% confidence intervals (CIs) were calculated using the Mantel–Haenszel method under a random-effects model to account for potential heterogeneity. Heterogeneity was quantified using the I^2^ statistic, with values interpreted as low (< 25%), moderate (25–75%), or high (> 75%). Statistical significance was set at *P* < 0.05. Publication bias was assessed through visual inspection of funnel plots; however, formal funnel plot asymmetry tests were not performed owing to the small number of studies (*n* < 10), which would result in inadequate statistical power. For all meta-analyses, delayed reoperation (> 48 h) served as the reference group. In forest plots, odds ratios > 1.0 indicate increased risk with early reoperation for adverse outcomes. All analyses were performed using Review Manager (RevMan) software, version 5.4.1 (Cochrane Collaboration, Nordic Cochrane Centre, Copenhagen, Denmark).

## Results

### Study selection and characteristics

**Fig. 1:**
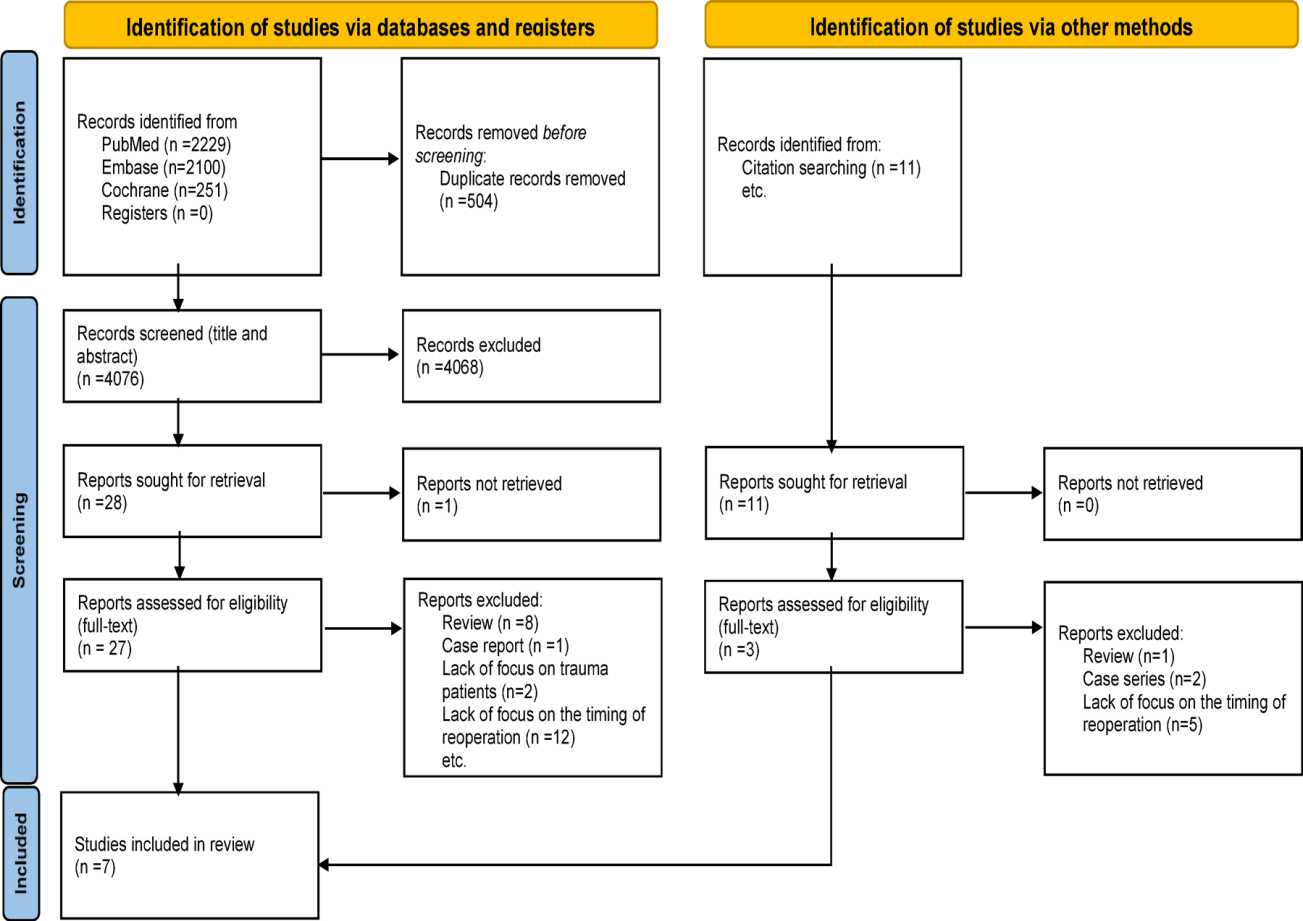
PRISMA flow diagram regarding the literature search. *PRISMA* preferred reporting items for systematic reviews and meta-analyses

A total of 4,580 records were identified through the database search. After removal of duplicates, 4,076 titles and abstracts were screened, and 27 full-text articles were assessed for eligibility. Seven studies met the inclusion criteria and were included in the quantitative analysis (Fig. 1). All included studies were retrospective cohort designs published between 1999 and 2024, with sample sizes ranging from 44 to 499 patients (total, *n* = 965). The study populations were predominantly young males (mean age: 28–44 years) with both penetrating and blunt trauma mechanisms (Table 3). Three studies [10, 12, 14] reported re-bleeding outcomes, four [10–13] reported mortality, and six [10–15] reported infection-related outcomes.

**Table 3:** Summary of detailed information about the included studies

Author	Country	Study design	Research period	Sample size	Patient type	Study setting	Follow-up duration	Outcomes
Jeong E et al. (2024)	South Korea	Retrospective, Single-center	2012–2021	101	Blunt trauma with DCL	Re-laparotomy timing: ≤48 h vs > 48 h	Until hospital discharge	Mortality Complication includes infection Primary fascia closure Hospital LOS, ICU LOS
Ordonez C et al. (2012)	Colombia	Retrospective, Single-center	2003–2010	121	Penetrating trauma with DCL	Packing duration: <1, 1–2, 2–3, > 3 days	30 days post-operation	Mortality Re-bleeding Complication includes infection Hospital LOS, ICU LOS
Kang B et al. (2021)	South Korea	Retrospective, Single-center	2011–2019	65	Trauma with perihepatic packing	Re-laparotomy timing: ≤48 h vs > 48 h	Until hospital discharge	Mortality Re-packing ICU LOS, ventilation day Complication includes infection
Nicol AJ et al. (2007)	South Africa	Retrospective, Single-center	1996–2004	72	Liver trauma with DCL	Re-laparotomy timing: 24 h vs 48 h vs 72 h	Until hospital discharge	Re-bleeding Complication includes infection
Caruso DM et al. (1999)	USA	Retrospective, Single-center	1988–1997	63	Liver trauma with DCL	Re-laparotomy timing: ≤36 h vs 36-72 h	Until hospital discharge	Mortality Re-bleeding Comlication Hospital LOS, ICU LOS
Pommerening MJ et al. (2014)	USA	Prospective, Multicenter	2010–2011	499	Trauma with DCL	Re-laparotomy timing: ≤48 h vs > 48 h	Until hospital discharge	Mortality Complication includes infection Primary fascia closure Hospital LOS, ICU LOS
Kim K et al. (2022)	South Korea	Retrospective, Single-center	2012–2021	44	Pelvic trauma with DCL	Re-operation timing: ≤48 h vs > 48 h	30 days post-operation	Surgical site infection

### Risk of bias assessment and quality assessment

The NOS scores ranged from 6 to 9 points, indicating moderate to high methodological quality. Most studies demonstrated adequate selection criteria and outcome assessment; however, comparability was often limited by a lack of adjustment for potential confounders. All included studies carried a moderate to high risk of bias because of their retrospective nature. Selection bias was present in all studies, as reoperation timing was determined by clinical judgment rather than randomisation. Five studies (62.5%) lacked clear definitions of infection outcomes, introducing potential detection bias.

The GRADE assessment indicated moderate-quality evidence for the primary outcome of re-bleeding and low- to very low-quality evidence for mortality and infection outcomes. The downgrading was primarily owing to the observational design, variability in outcome definitions, and imprecision arising from small sample sizes (Tables 1 and 2).

### Primary outcome: re-bleeding

**Fig. 2:**
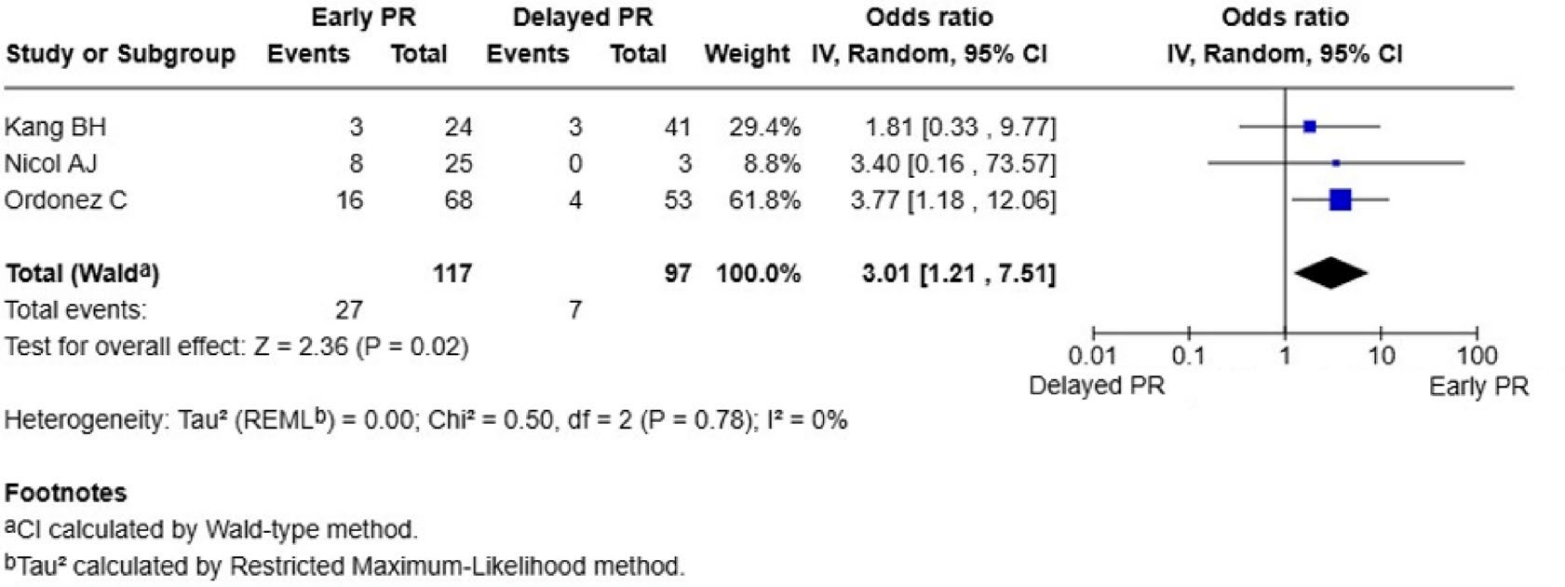
Forest plot of rebleeding rates: early versus delayed planned reoperation. PR, planned reoperation; CI, confidence interval. Participant numbers are outcome-specific and correspond to the studies reporting data for that outcome

Three studies (*n* = 258 patients) reported re-bleeding rates following planned reoperation [10, 12, 14]. The number of patients included in each meta-analysis differed by outcome because not all studies reported every endpoint. Early planned reoperation (≤ 48 h) was associated with significantly higher re-bleeding rates compared to delayed reoperation (>48 h). The pooled odds ratio was 3.01 (95% CI 1.21–7.51; *P* = 0.02), indicating that patients undergoing early reoperation had three-fold higher odds of re-bleeding. No statistical heterogeneity was detected (I^2^ = 0%, *P* = 0.78) (Fig. 2). One additional study, which used a 36-h threshold, reported similar results favouring delayed pack removal but was not included in the pooled analysis because of differing timing definitions [16].

### Secondary outcomes

**Fig. 3:**
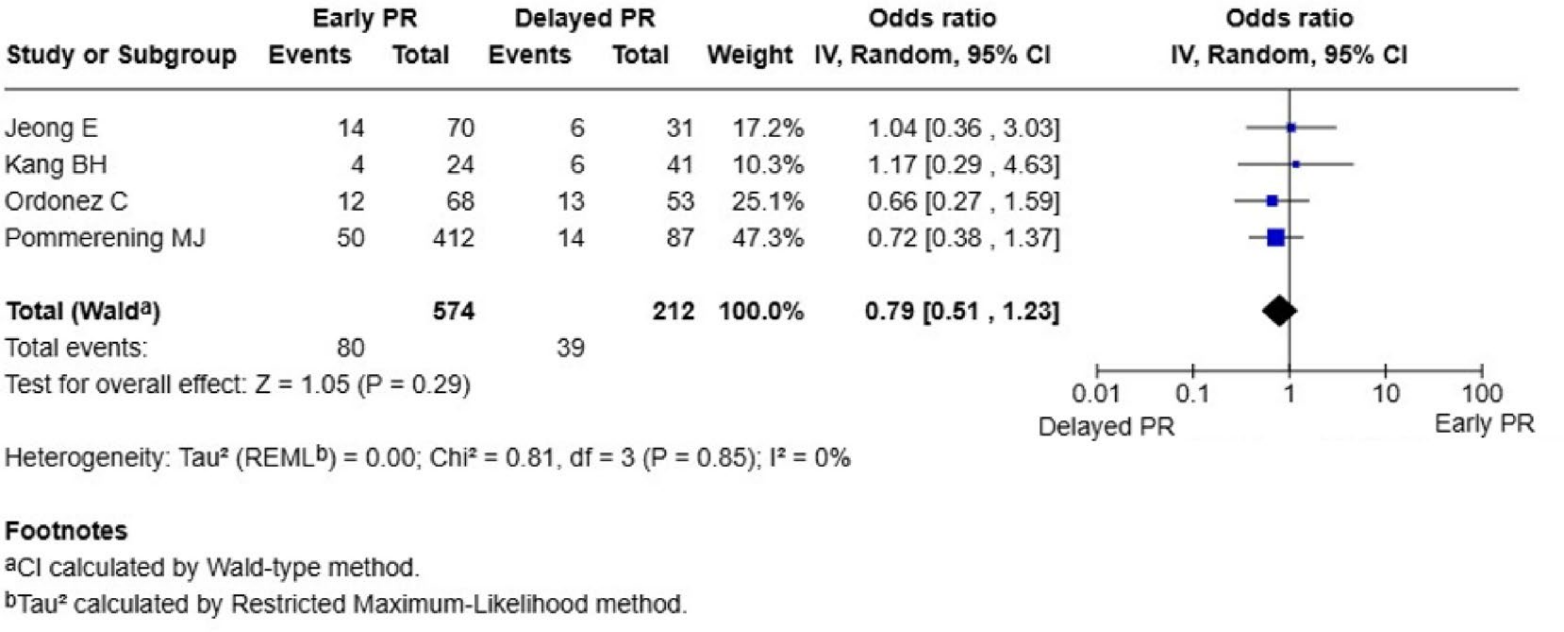
Forest plot of in-hospital mortality: early versus delayed planned reoperation. PR, planned reoperation; CI, confidence interval. Participant numbers are outcome-specific and correspond to the studies reporting data for that outcome

*In-hospital mortality*: Four studies (*n* = 786 patients) found no statistically significant difference in mortality between early and delayed planned reoperation groups (OR 0.79; 95% CI 0.51–1.23; *P* = 0.29) [10–13]. No heterogeneity was observed (I^2^ = 0%, *P* = 0.85) (Fig. 3).

**Fig. 4:**
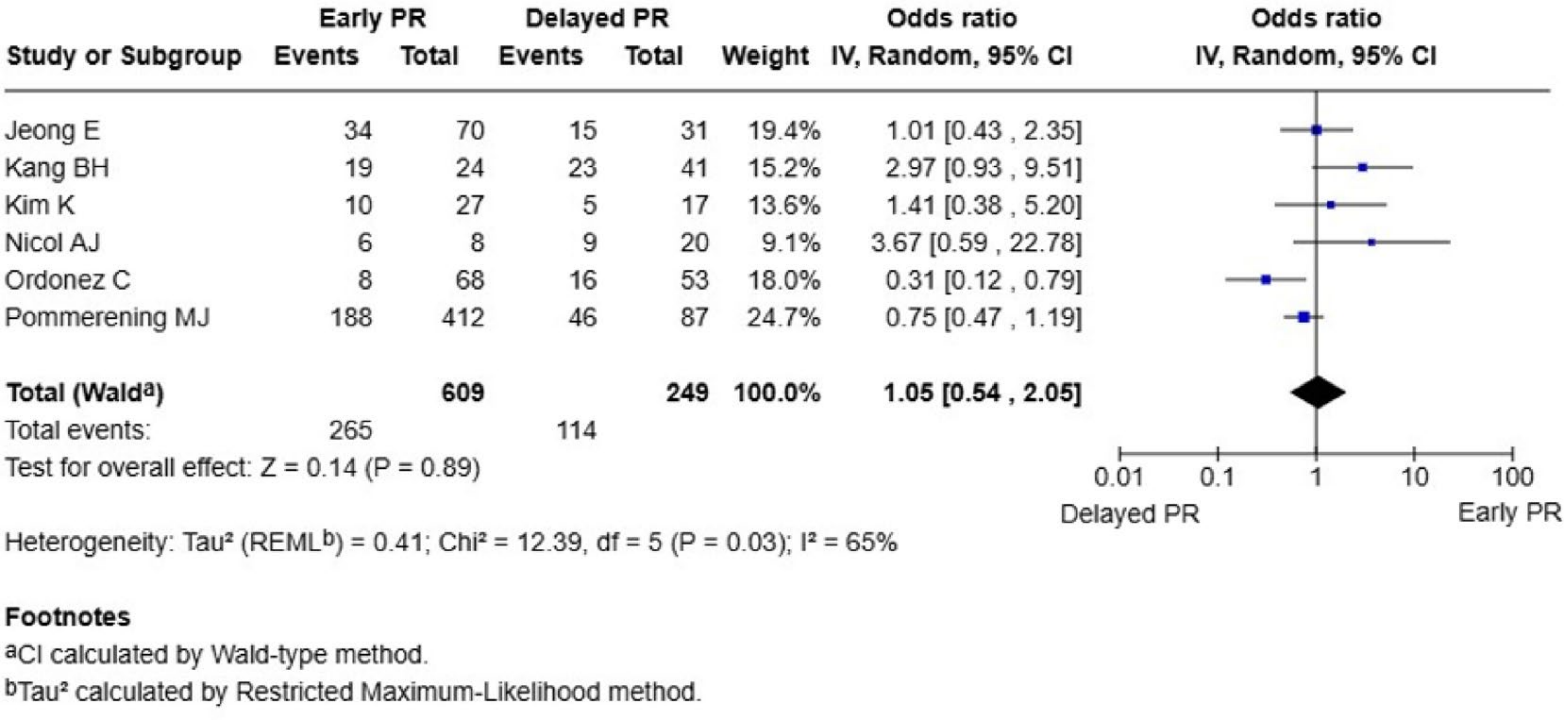
Forest plot of infection rates: early versus delayed planned reoperation. PR, planned reoperation; CI, confidence interval. Participant numbers are outcome-specific and correspond to the studies reporting data for that outcome

*Infection*: Six studies (*n* = 902 patients) reported no significant difference in infection rates between early and delayed planned reoperation groups (OR 1.05; 95% CI 0.54–2.05; *P* = 0.89). Moderate heterogeneity was present (I^2^ = 65%, *P* = 0.03) [10–15] (Fig. 4).

## Discussion

This meta-analysis of 965 patients with trauma from seven centres found that early reoperation carries three-fold higher odds of re-bleeding (OR 3.01) supports the physiological principle that hemostasis requires 48–72 h for stabilization. The findings in this study challenge the conventional 24- to 48-h window [8, 9] and support a more individualised approach, with timing tailored to each patient’s physiological status rather than adherence to rigid protocols.

The heterogeneity in DCS indications represents a fundamental consideration for interpreting our findings. The pathophysiological basis for reoperation timing differs substantially by indication. For hemorrhage-related DCS, the coagulation cascade requires 48–72 h for complete stabilization through fibrin cross-linking and clot retraction. Premature reoperation may disrupt this process, explaining our finding of increased re-bleeding with early intervention (OR 3.01). Nicol et al. specifically demonstrated this in hepatic injuries, where pack removal at 24 h resulted in 48% re-bleeding versus 6% after 48 h [14]. For contamination-related DCS, different considerations apply. Despite theoretical concerns about bacterial proliferation with delayed reoperation, our meta-analysis found no significant difference in infection rates (OR 1.05, 95% CI 0.54–2.05), possibly reflecting effective modern antimicrobial therapy and the importance of source control over timing alone. Future research should prioritize: (1) Pre-specified subgroup analyses by DCS indication, (2) Anatomical site-specific protocols, (3) Integration of physiological parameters for timing decisions, and (4) Development of indication-specific reoperation algorithms.

DCS is a staged surgical strategy for physiologically unstable patients with trauma and massive haemorrhage of the thorax, abdomen, or pelvis. It prioritises rapid haemostasis and physiological stabilisation, followed by planned reoperation to complete the definitive repair once the patient’s condition permits. Systematic reviews and multiple studies have demonstrated that DCS in exsanguinating trauma improves survival and clinical outcomes in patients with abdominal injuries [17–21]. Gauze packing has also been shown to be effective in managing severe pelvic haemorrhage [22–24], and intrathoracic packing can substantially reduce bleeding [25, 26].

The timing of reoperation and gauze removal should be determined by considering the patient’s condition, available resources, and the surgeon’s clinical judgment [27]. However, robust evidence and definitive guidelines remain lacking. Current recommendations are predominantly based on limited observational data and expert opinion, rather than high-quality prospective trials. Some authors advocate for planned reoperation within 24–48 h, arguing that early intervention may enhance haemostasis, reduce infection risk, and improve abdominal closure rates [8]. In contrast, reports from resource-limited or logistically constrained settings suggest that maintaining packing for more than 3 days can control haemorrhage without major complications [28].

Removing gauze or undertaking reoperation prematurely may increase the risk of re-bleeding because of incomplete haemostasis. For example, Kang et al. found no significant relationship between removal timing and the need for repacking [12], whereas Nicol et al. reported higher re-bleeding rates when packing was removed within 24 h [14]. Similarly, Ordoñez et al. observed substantially increased re-bleeding rates when removal occurred within 1 day, with progressive risk reduction as the duration increased [10]. Conversely, excessively delayed removal may lead to the adhesion of the gauze to injured organs, vessels, peritoneum, or bowel, potentially causing re-bleeding during extraction [28]. In the present analysis, re-bleeding rates were significantly lower when removal occurred after 48 h, indicating that timing may be a key determinant of re-bleeding risk.

Several previous studies have examined the relationship between reoperation timing or gauze removal and mortality, but most reported no statistically significant differences [11–13, 16]. Ordoñez et al. noted that prolonged packing was associated with reduced mortality from re-bleeding but increased mortality from intra-abdominal infection [10]. In our analysis, no mortality difference was observed between early and delayed groups, suggesting that mortality is likely influenced by factors other than reoperation timing alone.

With respect to infection, most studies similarly failed to demonstrate a significant association [11, 13–15]. However, Kang et al. reported a higher incidence of pneumonia when gauze was removed after 48 h [12], and Ordoñez et al. found that packing maintained for more than 3 days increased the risk of intra-abdominal infection by nearly threefold compared with packing maintained for 1–2 days [10]. Some authors have suggested that gauze packing may carry inherent infection risks, with removal within 96 h generally considered safe [11]. In the present analysis, no statistically significant association between removal timing and intra-abdominal infection was observed, although some heterogeneity arose from variations in the definitions of infection across studies.

Several studies have also explored the relationship between the timing of gauze removal and abdominal wall closure rates, with findings indicating that prolonged open abdomen duration reduces the likelihood of successful primary fascial closure. Early fascial closure is therefore recommended when feasible, as it may decrease mortality, complications, and hospital stay associated with an open abdomen [8, 29, 30, 31]. Although the current meta-analysis did not assess the association between reoperation timing and fascial closure success, this factor warrants consideration when determining optimal timing.

This study has several limitations. First, no randomised controlled trials were identified, and the analysis was based exclusively on cohort studies, which provide a lower level of evidence. This limitation, however, highlights the value of synthesising available observational data to guide clinical decision-making. Second, definitions of gauze removal timing varied among the included studies; nonetheless, a uniform 48-h cut-off was applied to enhance comparability. Third, infection definitions were heterogeneous, reflecting the diverse clinical presentations observed in practice.

Several systematic reviews and meta-analyses on DCS have been published, including evaluations of its indications in civilian trauma [18], analyses of its effectiveness in trauma patients [32], and comparisons of outcomes between DCS and traditional surgical approaches in non-trauma patients [33]. However, evidence on the impact of reoperation or gauze removal timing after DCS on outcomes such as mortality, rebleeding, and infection remains limited. This study addresses this gap by systematically analyzing the relationship between timing and these outcomes and provides a basis for future clinical decision-making.

## Conclusion

This meta-analysis indicates that delayed planned reoperation (> 48 h) after damage control surgery reduces the risk of re-bleeding without increasing mortality or infection rates. These findings support individualised timing based on patient physiology rather than rigid adherence to conventional 24- to 48-h protocols, particularly in those at high risk of bleeding. Well-designed prospective randomised trials are needed to confirm these results and to establish evidence-based timing strategies.

## Supplementary Information

Below is the link to the electronic supplementary material.

Supplementary Material 1

Supplementary Material 2

## Data Availability

All data extracted for this systematic review are included in this published article and its supplementary files. The protocol is registered and available at PROSPERO (CRD420251049990). Additional data extraction forms are available from the corresponding authors upon request.

## Abbreviations

CI: Confidence interval
DCS: Damage control surgery
EAST: Eastern Association for the Surgery of Trauma
GRADE: Grading of Recommendations Assessment, Development and Evaluation
ICU: Intensive Care Unit
I^2^: Inconsistency Index (statistical measure of heterogeneity)
MeSH: Medical subject headings
NOS: Newcastle–Ottawa scale
OR: Odds ratio
PRISMA: Preferred reporting items for systematic reviews and meta-analyses
PROSPERO: International prospective register of systematic reviews
WSES: World Society of Emergency Surgery
