# Supplementary material for: Timing of planned reoperation after damage control surgery in patients with trauma: a systematic review and meta-analysis
Source: World J Emerg Surg. 2025 Oct 29;20:82. doi: 10.1186/s13017-025-00657-9 (PMC12574027; doi:10.1186/s13017-025-00657-9)
Supplement: Supplementary file 1 — Supplementary Material 1 [file 13017_2025_657_MOESM1_ESM.docx]

| **Supplementary Digital Content 1: Detailed Search Strategy** | | | |  |  |
| --- | --- | --- | --- | --- | --- |
|  | | |  | |  |
| **PubMed** | | |  | |  |
| No. | Query | | | | Results |
| #12 | #11 AND ("English"[lang] OR "Korean"[lang]) | | | | **2,229** |
| #11 | (#10 NOT ("Animals"[mesh] NOT "Humans"[mesh])) NOT (("Adolescent"[mesh] OR "Child"[mesh] OR "Infant"[mesh]) NOT "Adult"[mesh]) | | | | 2,483 |
| #10 | #6 AND (#7 OR #8 OR #9) | | | | 2,601 |
| #9 | "Infections"[mesh] OR "Wound Infection"[mesh] OR "Surgical Wound Infection"[mesh] OR "Surgical Wound Infection/surgery"[mesh] OR "infection*"[tw] OR "infectious"[tw] | | | | 4,178,259 |
| #8 | "Hemorrhage"[mesh] OR "hemorrhag*"[tw] OR "haemorrhag*"[tw] OR "bleed*"[tw] OR "blood effusion*"[tw] OR "blood loss*"[tw] OR "rehemorrhag*"[tw] OR "rehaemorrhag*"[tw] OR "rebleed*"[tw] | | | | 783,943 |
| #7 | "Mortality"[mesh] OR "mortality"[Subheading] OR "Survival"[mesh] OR "mortalit*"[tw] OR "death"[tw] OR "fatalit*"[tw] OR "surviv*"[tw] OR "lethal outcome*"[tw] OR "fatal outcome*"[tw] OR "demise"[tw] OR "mors"[tw] | | | | 3,713,493 |
| #6 | #3 AND #4 AND #5 | | | | 4,760 |
| #5 | "Time Factors"[mesh] OR "timing"[tiab] OR "time factor*"[tiab] OR "timely"[tiab] OR "when"[ti] OR (("time to surgery"[tiab:~2] OR "time to surgeries"[tiab:~2] OR "time to surgical"[tiab:~2] OR "time to resurgery"[tiab:~2] OR "time to resurgeries"[tiab:~2] OR "time to resurgical"[tiab:~2] OR "time to re-surgery"[tiab:~2] OR "time to re-surgeries"[tiab:~2] OR "time to re-surgical"[tiab:~2] OR "time to operation"[tiab:~2] OR "time to operations"[tiab:~2] OR "time to operative"[tiab:~2] OR "time to reoperation"[tiab:~2] OR "time to reoperations"[tiab:~2] OR "time to reoperative"[tiab:~2] OR "time to re-operation"[tiab:~2] OR "time to re-operations"[tiab:~2] OR "time to re-operative"[tiab:~2] OR "time to second look"[tiab:~2] OR "time to 2nd look"[tiab:~2] OR "time to relook"[tiab:~2] OR "time to re-look"[tiab:~2] OR "time to reentry"[tiab:~2] OR "time to re-entry"[tiab:~2] OR "time to gauze removal"[tiab:~2] OR "time to gauze removals"[tiab:~2] OR "time to pack removal"[tiab:~2] OR "time to packed removal"[tiab:~2] OR "time to packing removal"[tiab:~2] OR "time to pack removals"[tiab:~2] OR "time to packed removals"[tiab:~2] OR "time to packing removals"[tiab:~2] OR "time to dressing removal"[tiab:~2] OR "time to dressing removals"[tiab:~2]) AND "time to"[tiab]) OR "how long"[tiab] OR "delayed surgery"[tiab:~1] OR "delayed surgeries"[tiab:~1] OR "delayed surgical"[tiab:~1] OR "delayed resurgery"[tiab:~1] OR "delayed resurgeries"[tiab:~1] OR "delayed resurgical"[tiab:~1] OR "delayed re-surgery"[tiab:~1] OR "delayed re-surgeries"[tiab:~1] OR "delayed re-surgical"[tiab:~1] OR "delayed operation"[tiab:~1] OR "delayed operations"[tiab:~1] OR "delayed operative"[tiab:~1] OR "delayed reoperation"[tiab:~1] OR "delayed reoperations"[tiab:~1] OR "delayed reoperative"[tiab:~1] OR "delayed re-operation"[tiab:~1] OR "delayed re-operations"[tiab:~1] OR "delayed re-operative"[tiab:~1] OR "delayed second look"[tiab:~1] OR "delayed 2nd look"[tiab:~1] OR "delayed relook"[tiab:~1] OR "delayed re-look"[tiab:~1] OR "delayed reentry"[tiab:~1] OR "delayed re-entry"[tiab:~1] OR "delayed gauze removal"[tiab:~1] OR "delayed gauze removals"[tiab:~1] OR "delayed pack removal"[tiab:~1] OR "delayed packed removal"[tiab:~1] OR "delayed packing removal"[tiab:~1] OR "delayed pack removals"[tiab:~1] OR "delayed packed removals"[tiab:~1] OR "delayed packing removals"[tiab:~1] OR "delayed dressing removal"[tiab:~1] OR "delayed dressing removals"[tiab:~1] OR "delay surgery"[tiab:~1] OR "delay surgeries"[tiab:~1] OR "delay surgical"[tiab:~1] OR "delay resurgery"[tiab:~1] OR "delay resurgeries"[tiab:~1] OR "delay resurgical"[tiab:~1] OR "delay re-surgery"[tiab:~1] OR "delay re-surgeries"[tiab:~1] OR "delay re-surgical"[tiab:~1] OR "delay operation"[tiab:~1] OR "delay operations"[tiab:~1] OR "delay operative"[tiab:~1] OR "delay reoperation"[tiab:~1] OR "delay reoperations"[tiab:~1] OR "delay reoperative"[tiab:~1] OR "delay re-operation"[tiab:~1] OR "delay re-operations"[tiab:~1] OR "delay re-operative"[tiab:~1] OR "delay second look"[tiab:~1] OR "delay 2nd look"[tiab:~1] OR "delay relook"[tiab:~1] OR "delay re-look"[tiab:~1] OR "delay reentry"[tiab:~1] OR "delay re-entry"[tiab:~1] OR "delay gauze removal"[tiab:~1] OR "delay gauze removals"[tiab:~1] OR "delay pack removal"[tiab:~1] OR "delay packed removal"[tiab:~1] OR "delay packing removal"[tiab:~1] OR "delay pack removals"[tiab:~1] OR "delay packed removals"[tiab:~1] OR "delay packing removals"[tiab:~1] OR "delay dressing removal"[tiab:~1] OR "delay dressing removals"[tiab:~1] OR "delays surgery"[tiab:~1] OR "delays surgeries"[tiab:~1] OR "delays surgical"[tiab:~1] OR "delays resurgery"[tiab:~1] OR "delays resurgeries"[tiab:~1] OR "delays resurgical"[tiab:~1] OR "delays re-surgery"[tiab:~1] OR "delays re-surgeries"[tiab:~1] OR "delays re-surgical"[tiab:~1] OR "delays operation"[tiab:~1] OR "delays operations"[tiab:~1] OR "delays operative"[tiab:~1] OR "delays reoperation"[tiab:~1] OR "delays reoperations"[tiab:~1] OR "delays reoperative"[tiab:~1] OR "delays re-operation"[tiab:~1] OR "delays re-operations"[tiab:~1] OR "delays re-operative"[tiab:~1] OR "delays second look"[tiab:~1] OR "delays 2nd look"[tiab:~1] OR "delays relook"[tiab:~1] OR "delays re-look"[tiab:~1] OR "delays reentry"[tiab:~1] OR "delays re-entry"[tiab:~1] OR "delays gauze removal"[tiab:~1] OR "delays gauze removals"[tiab:~1] OR "delays pack removal"[tiab:~1] OR "delays packed removal"[tiab:~1] OR "delays packing removal"[tiab:~1] OR "delays pack removals"[tiab:~1] OR "delays packed removals"[tiab:~1] OR "delays packing removals"[tiab:~1] OR "delays dressing removal"[tiab:~1] OR "delays dressing removals"[tiab:~1] | | | | 1,558,490 |
| #4 | "Second-Look Surgery"[mesh] OR "Reoperation"[mesh] OR ("Postoperative Care"[mesh] AND "surgery"[subheading]) OR (("second look"[ti] OR "2nd look"[ti] OR "relook"[ti] OR "re-look"[ti] OR "reentry"[ti] OR "re-entry"[ti]) AND ("surger*"[ti] OR "surgical"[ti] OR "operati*"[ti])) OR (("followup surgery"[ti:~1] OR "followup surgeries"[ti:~1] OR "followup surgical"[ti:~1] OR "followup operation"[ti:~1] OR "followup operative"[ti:~1] OR "follow up surgery"[ti:~1] OR "follow up surgeries"[ti:~1] OR "follow up surgical"[ti:~1] OR "follow up operation"[ti:~1] OR "follow up operative"[ti:~1]) NOT ("followup stud*"[ti] OR "follow up stud*"[ti] OR "follow up after"[ti])) OR "second look surg*"[tiab] OR "2nd look surg*"[tiab] OR "relook surg*"[tiab] OR "re look surg*"[tiab] OR "reentry surg*"[tiab] OR "re entry surg*"[tiab] OR "follow up surger*"[tiab] OR "follow up surgical"[tiab] OR "surg* followup"[tiab] OR "surg* follow up"[tiab] OR "second look operati*"[tiab] OR "relook operati*"[tiab] OR "reentry operati*"[tiab] OR "re entry operati*"[tiab] OR "operati* follow up"[tiab] OR "resurg*"[tiab] OR "re-surg*"[tiab] OR "reoperat*"[tiab] OR "re-operati*"[tiab] OR "packing"[ti] OR "gauze remov*"[tiab] OR "pack* remov*"[tiab] OR "pack removal"[tiab:~3] OR "packed removal"[tiab:~3] OR "packing removal"[tiab:~3] OR "pack removals"[tiab:~3] OR "packed removals"[tiab:~3] OR "packing removals"[tiab:~3] OR "dressing* remov*"[tiab] OR "re-laparotom*"[tiab] OR "relaparotom*"[tiab] | | | | 199,418 |
| #3 | #1 OR #2 | | | | 1,911,414 |
| #2 | "Wounds and Injuries"[mesh] OR "Trauma Centers"[mesh] OR "Acute Care Surgery"[mesh] OR "Critical Care"[mesh] OR "trauma*"[ti] OR "injury"[ti] OR "injuries"[ti] OR "reinjur*"[ti] OR "wounds"[ti] OR "acute care surg*"[tiab] OR "surgical acute care"[tiab] OR "emergen*"[ti] OR "emergen* surg*"[tiab] OR "emergen* general surg*"[tiab] OR "critical care surg*"[tiab] OR "surgical critical care"[tiab] OR "trauma* surg*"[tiab] OR "trauma* care surg*"[tiab] OR "surgical trauma*"[tiab] OR "truncal injur*"[tiab] OR "abdominal*"[ti] OR "abdomen"[ti] OR "digestive system*"[ti] OR "laparo*"[ti] OR "pelvic"[ti] OR "pelvis"[ti] OR "thorax"[ti] OR "thoracic"[ti] OR "cardiothoracic"[ti] OR "chest"[ti] OR (("Wounds and Injuries"[majr] OR "Trauma Centers"[majr] OR "Acute Care Surgery"[majr] OR "Critical Care"[majr]) AND ("truncal injur*"[tw] OR "abdominal*"[tw] OR "abdomen"[tw] OR "digestive system*"[tw] OR "laparo*"[tw] OR "pelvic"[tw] OR "pelvis"[tw] OR "thorax"[tw] OR "thoracic"[tw] OR "cardiothoracic"[tw] OR "chest"[tw])) | | | | 1,910,169 |
| #1 | ("damage control*"[ti] AND ("surg*"[ti] OR "strateg*"[ti] OR "procedure*"[ti] OR "intervention*"[ti] OR "Laparotomy"[mesh] OR "laparotom*"[ti] OR "Resuscitation"[mesh] OR "resuscitation*"[ti])) OR "damage control*"[tiab] OR "damage control surg*"[tiab] OR "damage control surgery"[tiab:~3] OR "damage control surgeries"[tiab:~3] OR "damage control surgical"[tiab:~3] OR "damage control strateg*"[tiab] OR "damage control strategy"[tiab:~3] OR "damage control strategies"[tiab:~3] OR "damage control procedure"[tiab:~3] OR "damage control procedures"[tiab:~3] OR "damage control intervention"[tiab:~3] OR "damage control interventions"[tiab:~3] OR "damage control interventional"[tiab:~3] OR "damage control laparotomy"[tiab:~3] OR "damage control laparotomies"[tiab:~3] OR "damage control resuscitation"[tiab:~3] OR "damage control resuscitations"[tiab:~3] OR (("DCS"[tiab] OR "DCSs"[tiab]) AND "damag*"[tiab] AND "control*"[tiab]) OR "damage surg*"[tiab] | | | | 3,881 |
|  | |  | | |  |
| **Embase** | |  | | |  |
| No. | Query | | | | Results |
| #12 | #11 AND ([english]/lim OR [korean]/lim) | | | | **2,100** |
| #11 | #10 NOT (('animal'/exp OR 'nonhuman'/exp) NOT 'human'/exp) NOT (([embryo]/lim OR [fetus]/lim OR [newborn]/lim OR [infant]/lim OR [child]/lim OR [preschool]/lim OR [school]/lim OR [adolescent]/lim) NOT ([adult]/lim OR [young adult]/lim OR [middle aged]/lim OR [aged]/lim OR [very elderly]/lim)) | | | | 2,185 |
| #10 | #6 AND (#7 OR #8 OR #9) | | | | 2,381 |
| #9 | ('infection'/exp OR 'infectious complication'/exp OR 'wound infection'/exp OR 'device infection'/exp OR 'infection*':ti,ab,kw OR 'infectious':ti,ab,kw) | | | | 5,828,552 |
| #8 | ('bleeding'/exp OR 'hemorrhag*':ti,ab,kw OR 'haemorrhag*':ti,ab,kw OR 'bleed*':ti,ab,kw OR 'blood effusion*':ti,ab,kw OR 'blood loss*':ti,ab,kw OR 'rehemorrhag*':ti,ab,kw OR 'rehaemorrhag*':ti,ab,kw OR 'rebleed*':ti,ab,kw) | | | | 1,601,917 |
| #7 | ('mortality'/exp OR 'survival'/exp OR 'mortalit*':ti,ab,kw OR 'death':ti,ab,kw OR 'fatalit*':ti,ab,kw OR 'surviv*':ti,ab,kw OR 'lethal outcome*':ti,ab,kw OR 'fatal outcome*':ti,ab,kw OR 'demise':ti,ab,kw OR 'mors':ti,ab,kw) | | | | 5,318,267 |
| #6 | #3 AND #4 AND #5 | | | | 3,647 |
| #5 | ('time factor'/exp OR 'timing':ti,ab,kw OR 'time factor*':ti,ab,kw OR 'timely':ti,ab,kw OR 'when':ti,kw OR (('time to' NEXT/3 ('surger*' OR 'surgical' OR 'resurger*' OR 'resurgical' OR 're-surger*' OR 're-surgical' OR 'operation*' OR 'operative' OR 're-operation*' OR 're-operative' OR 'second look' OR '2nd look' OR 'relook' OR 're-look' OR 'reentry' OR 're-entry' OR 'gauze removal*' OR 'pack* removal*' OR 'dressing* removal*')):ti,ab,kw) OR 'how long':ti,ab,kw OR (('delay*' NEXT/2 ('surger*' OR 'surgical' OR 'resurger*' OR 'resurgical' OR 're-surger*' OR 're-surgical' OR 'operation*' OR 'operative' OR 're-operation*' OR 're-operative' OR 'second look' OR '2nd look' OR 'relook' OR 're-look' OR 'reentry' OR 're-entry' OR 'gauze removal*' OR 'pack* removal*' OR 'dressing* removal*')):ti,ab,kw)) | | | | 642,126 |
| #4 | ('second look surgery'/exp OR 'reoperation'/exp OR ('postoperative care'/exp AND 'surgery'/exp) OR (('second look':ti,kw OR '2nd look':ti,kw OR 'relook':ti,kw OR 're-look':ti,kw OR 'reentry':ti,kw OR 're-entry':ti,kw) AND ('surger*':ti,kw OR 'surgical':ti,kw OR 'operati*':ti,kw)) OR (((('followup' OR 'follow up') NEXT/2 ('surger*' OR 'surgical' OR 'operation*' OR 'operative')):ti,kw) NOT ('followup stud*':ti,kw OR 'follow up stud*':ti,kw OR 'follow up after':ti,kw)) OR 'second look surg*':ti,ab,kw OR '2nd look surg*':ti,ab,kw OR 'relook surg*':ti,ab,kw OR 're look surg*':ti,ab,kw OR 'reentry surg*':ti,ab,kw OR 're entry surg*':ti,ab,kw OR 'follow up surger*':ti,ab,kw OR 'follow up surgical':ti,ab,kw OR 'surg* followup':ti,ab,kw OR 'surg* follow up':ti,ab,kw OR 'second look operati*':ti,ab,kw OR 'relook operati*':ti,ab,kw OR 'reentry operati*':ti,ab,kw OR 're entry operati*':ti,ab,kw OR 'operati* follow up':ti,ab,kw OR 'resurg*':ti,ab,kw OR 're-surg*':ti,ab,kw OR 'reoperat*':ti,ab,kw OR 're-operati*':ti,ab,kw OR 'packing':ti,kw OR 'gauze remov*':ti,ab,kw OR 'pack* remov*':ti,ab,kw OR ((('pack' OR 'packed' OR 'packing') NEAR/3 'removal*'):ti,ab,kw) OR 'dressing* remov*':ti,ab,kw OR 're-laparotom*':ti,ab,kw OR 'relaparotom*':ti,ab,kw) | | | | 314,034 |
| #3 | #1 OR #2 | | | | 4,251,952 |
| #2 | ('injury'/exp OR 'emergency health service'/exp OR 'acute care surgery'/exp OR 'trauma*':ti,kw OR 'injury':ti,kw OR 'injuries':ti,kw OR 'reinjur*':ti,kw OR 'wounds':ti,kw OR 'acute care surg*':ti,ab,kw OR 'surgical acute care':ti,ab,kw OR 'emergen*':ti,kw OR 'emergen* surg*':ti,ab,kw OR 'emergen* general surg*':ti,ab,kw OR 'critical care surg*':ti,ab,kw OR 'surgical critical care':ti,ab,kw OR 'trauma* surg*':ti,ab,kw OR 'trauma* care surg*':ti,ab,kw OR 'surgical trauma*':ti,ab,kw OR 'truncal injur*':ti,ab,kw OR 'abdominal*':ti,kw OR 'abdomen':ti,kw OR 'digestive system*':ti,kw OR 'laparo*':ti,kw OR 'pelvic':ti,kw OR 'pelvis':ti,kw OR 'thorax':ti,kw OR 'thoracic':ti,kw OR 'cardiothoracic':ti,kw OR 'chest':ti,kw OR (('injury'/exp/mj OR 'emergency health service'/exp/mj OR 'acute care surgery'/exp/mj) AND ('truncal injur*':ti,ab,kw OR 'abdominal*':ti,ab,kw OR 'abdomen':ti,ab,kw OR 'digestive system*':ti,ab,kw OR 'laparo*':ti,ab,kw OR 'pelvic':ti,ab,kw OR 'pelvis':ti,ab,kw OR 'thorax':ti,ab,kw OR 'thoracic':ti,ab,kw OR 'cardiothoracic':ti,ab,kw OR 'chest':ti,ab,kw))) | | | | 4,250,703 |
| #1 | ('damage control'/exp OR 'damage control surgery'/exp OR 'damage control resuscitation'/exp OR 'damage control laparotomy'/exp OR 'damage control orthopedics'/exp OR ('damage control*':ti,kw AND ('surg*':ti,kw OR 'strateg*':ti,kw OR 'procedure*':ti,kw OR 'intervention*':ti,kw OR 'laparotomy'/exp OR 'laparotom*':ti,kw OR 'resuscitation'/exp OR 'resuscitation*':ti,kw)) OR 'damage control*':ti,ab,kw OR 'damage control surg*':ti,ab,kw OR (('damage control' NEXT/3 ('surger*' OR 'surgical' OR 'strateg*' OR 'procedure*' OR 'intervention*' OR 'laparotom*' OR 'resuscitat*')):ti,ab,kw) OR (('dcs':ti,ab,kw OR 'dcss':ti,ab,kw) AND 'damag*':ti,ab,kw AND 'control*':ti,ab,kw) OR 'damage surg*':ti,ab,kw) | | | | 5,400 |
|  | |  | | |  |
| **Cochrane Library** | | | | |  |
| No. | Query | | | | Results |
| #15 | #13 OR #14 | | | | **251** |
| #14 | #12 AND Korean:la | | | | - |
| #13 | #12 AND English:la | | | | 251 |
| #12 | #11 NOT (([mh "Adolescent"] OR [mh "Child"] OR [mh "Infant"]) NOT [mh "Adult"]) | | | | 259 |
| #11 | #10 NOT ([mh "Animals"] NOT [mh "Humans"]) | | | | 267 |
| #10 | #6 AND (#7 OR #8 OR #9) | | | | 267 |
| #9 | [mh "Infections"] OR [mh "Wound Infection"] OR [mh "Surgical Wound Infection"] OR [mh "Surgical Wound Infection"/SU] OR infection*:ti,ab,kw OR infectious:ti,ab,kw | | | | 203,583 |
| #8 | [mh "Hemorrhage"] OR hemorrhag*:ti,ab,kw OR haemorrhag*:ti,ab,kw OR bleed*:ti,ab,kw OR (blood NEXT effusion*):ti,ab,kw OR (blood NEXT loss*):ti,ab,kw OR rehemorrhag*:ti,ab,kw OR rehaemorrhag*:ti,ab,kw OR rebleed*:ti,ab,kw | | | | 101,302 |
| #7 | [mh "Mortality"] OR [mh /MO] OR [mh "Survival"] OR mortalit*:ti,ab,kw OR death:ti,ab,kw OR fatalit*:ti,ab,kw OR surviv*:ti,ab,kw OR (lethal NEXT outcome*):ti,ab,kw OR (fatal NEXT outcome*):ti,ab,kw OR demise:ti,ab,kw OR mors:ti,ab,kw | | | | 284,514 |
| #6 | #3 AND #4 AND #5 | | | | 471 |
| #5 | [mh "Time Factors"] OR timing:ti,ab,kw OR (time NEXT factor*):ti,ab,kw OR timely:ti,ab,kw OR when:ti,kw OR ((time NEXT to) NEXT/3 (surger* OR surgical OR resurger* OR resurgical OR (re NEXT surger*) OR (re NEXT surgical) OR operation* OR operative OR (re NEXT operation*) OR (re NEXT operative) OR (second NEXT look) OR (2nd NEXT look) OR relook OR (re NEXT look) OR reentry OR (re NEXT entry) OR (gauze NEXT removal*) OR (pack* NEXT removal*) OR (dressing* NEXT removal*))):ti,ab,kw OR (how NEXT long):ti,ab,kw OR (delay* NEXT/2 (surger* OR surgical OR resurger* OR resurgical OR (re NEXT surger*) OR (re NEXT surgical) OR operation* OR operative OR (re NEXT operation*) OR (re NEXT operative) OR (second NEXT look) OR (2nd NEXT look) OR relook OR (re NEXT look) OR reentry OR (re NEXT entry) OR (gauze NEXT removal*) OR (pack* NEXT removal*) OR (dressing* NEXT removal*))):ti,ab,kw | | | | 111,898 |
| #4 | [mh "Second-Look Surgery"] OR [mh "Reoperation"] OR ([mh "Postoperative Care"] AND [mh /SU]) OR (((second NEXT look):ti,kw OR (2nd NEXT look):ti,kw OR relook:ti,kw OR (re NEXT look):ti,kw OR reentry:ti,kw OR (re NEXT entry):ti,kw) AND (surger*:ti,kw OR surgical:ti,kw OR operati*:ti,kw)) OR (((followup OR (follow NEXT up)) NEXT/2 (surger* OR surgical OR operation* OR operative)):ti,kw NOT ((followup NEXT stud*):ti,kw OR (follow NEXT up NEXT stud*):ti,kw OR (follow NEXT up NEXT after):ti,kw)) OR (second NEXT look NEXT surg*):ti,ab,kw OR (2nd NEXT look NEXT surg*):ti,ab,kw OR (relook NEXT surg*):ti,ab,kw OR (re NEXT look NEXT surg*):ti,ab,kw OR (reentry NEXT surg*):ti,ab,kw OR (re NEXT entry NEXT surg*):ti,ab,kw OR (follow NEXT up NEXT surger*):ti,ab,kw OR (follow NEXT up NEXT surgical):ti,ab,kw OR (surg* NEXT followup):ti,ab,kw OR (surg* NEXT follow NEXT up):ti,ab,kw OR (second NEXT look NEXT operati*):ti,ab,kw OR (relook NEXT operati*):ti,ab,kw OR (reentry NEXT operati*):ti,ab,kw OR (re NEXT entry NEXT operati*):ti,ab,kw OR (operati* NEXT follow NEXT up):ti,ab,kw OR resurg*:ti,ab,kw OR (re NEXT surg*):ti,ab,kw OR reoperat*:ti,ab,kw OR (re NEXT operati*):ti,ab,kw OR packing:ti,kw OR (gauze NEXT remov*):ti,ab,kw OR (pack* NEXT remov*):ti,ab,kw OR ((pack OR packed OR packing) NEAR/3 removal*):ti,ab,kw OR (dressing* NEXT remov*):ti,ab,kw OR (re NEXT laparotom*):ti,ab,kw OR relaparotom*:ti,ab,kw | | | | 16,032 |
| #3 | #1 OR #2 | | | | 191,522 |
| #2 | [mh "Wounds and Injuries"] OR [mh "Trauma Centers"] OR [mh "Acute Care Surgery"] OR [mh "Critical Care"] OR trauma*:ti,kw OR injury:ti,kw OR injuries:ti,kw OR reinjur*:ti,kw OR wounds:ti,kw OR (acute NEXT care NEXT surg*):ti,ab,kw OR (surgical NEXT acute NEXT care):ti,ab,kw OR emergen*:ti,kw OR (emergen* NEXT surg*):ti,ab,kw OR (emergen* NEXT general NEXT surg*):ti,ab,kw OR (critical NEXT care NEXT surg*):ti,ab,kw OR (surgical NEXT critical NEXT care):ti,ab,kw OR (trauma* NEXT surg*):ti,ab,kw OR (trauma* NEXT care NEXT surg*):ti,ab,kw OR (surgical NEXT trauma*):ti,ab,kw OR (truncal NEXT injur*):ti,ab,kw OR abdominal*:ti,kw OR abdomen:ti,kw OR (digestive NEXT system*):ti,kw OR laparo*:ti,kw OR pelvic:ti,kw OR pelvis:ti,kw OR thorax:ti,kw OR thoracic:ti,kw OR cardiothoracic:ti,kw OR chest:ti,kw OR (([mh "Wounds and Injuries"[mj]] OR [mh "Trauma Centers"[mj]] OR [mh "Acute Care Surgery"[mj]] OR [mh "Critical Care"[mj]]) AND ((truncal NEXT injur*):ti,ab,kw OR abdominal*:ti,ab,kw OR abdomen:ti,ab,kw OR (digestive NEXT system*):ti,ab,kw OR laparo*:ti,ab,kw OR pelvic:ti,ab,kw OR pelvis:ti,ab,kw OR thorax:ti,ab,kw OR thoracic:ti,ab,kw OR cardiothoracic:ti,ab,kw OR chest:ti,ab,kw)) | | | | 191,342 |
| #1 | ((damage NEXT control*):ti,kw AND (surg*:ti,kw OR strateg*:ti,kw OR procedure*:ti,kw OR intervention*:ti,kw OR [mh "Laparotomy"] OR laparotom*:ti,kw OR [mh "Resuscitation"] OR resuscitation*:ti,kw)) OR (damage NEXT control*):ti,ab,kw OR (damage NEXT control NEXT surg*):ti,ab,kw OR ((damage NEXT control) NEXT/3 (surger* OR surgical OR strateg* OR procedure* OR intervention* OR laparotom* OR resuscitat*)):ti,ab,kw OR ((DCS:ti,ab,kw OR DCSs:ti,ab,kw) AND damag*:ti,ab,kw AND control*:ti,ab,kw) OR (damage NEXT surg*):ti,ab,kw | | | | 298 |
