# Supplementary material for: Timing of planned reoperation after damage control surgery in patients with trauma: a systematic review and meta-analysis
Source: World J Emerg Surg. 2025 Oct 29;20:82. doi: 10.1186/s13017-025-00657-9 (PMC12574027; doi:10.1186/s13017-025-00657-9)
Supplement: Supplementary file 2 — Supplementary Material 2 [file 13017_2025_657_MOESM2_ESM.docx]

**Supplementary Table S1: Definitions of Infection Outcomes Across Included Studies**

| **Study** | **Infection Type Measured** | **Specific Definition** | **Diagnostic Criteria** | **Time to Diagnosis** |
| --- | --- | --- | --- | --- |
| **Ordonez C et al. [7] (2012)** | Intra-abdominal infection | • Intra-abdominal abscess requiring drainage  • Positive peritoneal fluid culture | • CT confirmation  • Clinical signs (fever >38°C, leukocytosis)  • Positive culture | Within 30 days |
| **Nicol AJ et al. [10] (2007)** | Septic complications | • Intra-abdominal sepsis  • Bile leaks  • Abscess formation | • Clinical sepsis criteria  • Relaparotomy findings  • Radiological confirmation | During hospital stay (mean 2.4 days for pack removal) |
| **Caruso DM et al. [11] (1999)** | Liver-related complications | • Biliary leak  • Intra-abdominal abscess | • Reoperation findings  • Drainage requirement  • Clinical deterioration | During hospital stay |
| **Jeong E et al. [8] (2024)** | Overall complications | • Wound infection (34.3%)  • Intra-abdominal abscess (14.3%)  • Pneumonia (18.6%) | • CDC criteria for SSI  • Positive cultures  • Clinical/radiological findings | During hospital stay |
| **Kang B et al. [9] (2021)** | Sepsis and VAP | • Clinical sepsis  • Ventilator-associated pneumonia | • SIRS criteria + source  • Positive sputum culture  • New infiltrate on CXR | During ICU stay |
| **Pommerening MJ et al. [13] (2014)** | Abdominal complications | • Abscess/fluid collection  • Enteric fistula  • Fascial dehiscence | • Reoperation findings  • CT confirmation  • Clinical deterioration | Until hospital discharge |
| **Kim K et al. [14] (2022)** | Surgical site infection | • Deep SSI at PPP site  • Organ/space SSI | • CDC criteria  • Purulent drainage  • Positive wound culture  • Reoperation for infection | Mean 8.1±3.9 days post-PPP |
